# Primary Tuberculosis of the Cervix Simulating Carcinoma

**Published:** 1902-04-19

**Authors:** 


### PRIMARY TUBERCULOSIS OF THE CERVIX SIMULATING CARCINOMA.

At the last meeting of the Obstetrical Society, Dr. Lewers described a case of primary tuberculosis of the cervix simulating cancer, which he had treated by vaginal hysterectomy. The patient was a married woman aged 36, who had never been pregnant. The symptoms of which she complained strongly suggested cancer, and on vaginal examination the condition of the vaginal portion of the cervix seemed identical with what is found in many cases of cancer. It appeared to be the seat of a "growth" which was friable and bled easily on examination. The uterus was freely movable. Vaginal hysterectomy was performed by Dr. Lewers, and the patient made a good recovery. On examination of the specimen, however, no evidence of cancer was found, the structure being tuberculous. From the cases mentioned by various speakers in the discussion on this paper, it is evident that tuberculosis of the cervix is not quite so great a rarity as some have imagined. It is evident, also, that in some cases it may be mistaken for cancer, which perhaps may explain some of the instances of which one hears in which cancer of the cervix is said to have been cured by what appears to have been a very moderate amount of scraping or cauterisation.

